# Human-Like Eukaryotic Translation Initiation Factor 3 from *Neurospora crassa*

**DOI:** 10.1371/journal.pone.0078715

**Published:** 2013-11-08

**Authors:** M. Duane Smith, Yu Gu, Jordi Querol-Audí, Jacob M. Vogan, Adam Nitido, Jamie H. D. Cate

**Affiliations:** 1 Department of Molecular and Cell Biology, University of California, Berkeley, California, United States of America; 2 Department of Chemistry, University of California, Berkeley, California, United States of America; 3 Physical Biosciences Division, Lawrence Berkeley National Laboratory, Berkeley, California, United States of America; University of British Columbia, Canada

## Abstract

Eukaryotic translation initiation factor 3 (eIF3) is a key regulator of translation initiation, but its *in vivo* assembly and molecular functions remain unclear. Here we show that eIF3 from *Neurospora crassa* is structurally and compositionally similar to human eIF3. *N. crassa* eIF3 forms a stable 12-subunit complex linked genetically and biochemically to the 13^th^ subunit, eIF3j, which in humans modulates mRNA start codon selection. Based on *N. crassa* genetic analysis, most subunits in eIF3 are essential. Subunits that can be deleted (e, h, k and l) map to the right side of the eIF3 complex, suggesting that they may coordinately regulate eIF3 function. Consistent with this model, subunits eIF3k and eIF3l are incorporated into the eIF3 complex as a pair, and their insertion depends on the presence of subunit eIF3h, a key regulator of vertebrate development. Comparisons to other eIF3 complexes suggest that eIF3 assembles around an eIF3a and eIF3c dimer, which may explain the coordinated regulation of human eIF3 levels. Taken together, these results show that *Neurospora crassa* eIF3 provides a tractable system for probing the structure and function of human-like eIF3 in the context of living cells.

## Introduction

The regulation of protein synthesis in eukaryotes occurs predominantly during translation initiation. Translation initiation in eukaryotes is regulated by a number of eukaryotic initiation factors (eIFs) whose specific roles in this process remain unclear. In humans, eIF3 is the largest eIF, consisting of 13 non-identical protein subunits named eIF3a through eIF3m [1]. During cap-dependent translation, eIF3 functions as a structural scaffold for other eIFs and is crucial in the formation of the translation pre-initiation complex (PIC) [2], [3]. Similarly, eIF3 is required for genomic RNA recruitment to the small ribosomal subunit during viral internal ribosome entry site (IRES)-dependent translation [2], [4], [5], [6]. Notably, altered expression levels of many subunits within eIF3–including eIF3a, b, c, e, f, h and m–have been linked to various cancers, although their roles in oncogenesis are not understood [7]. In zebrafish and worms, eIF3 subunits have been tied to developmental pathways that may require eIF3 to specifically recruit mRNAs to PICs [8], [9], [10]. Although the overall architecture of human eIF3 has recently been described [11], the specific functions of its subunits and its *in vivo* assembly pathway remain unclear [3], [11], [12].

**Figure 1:**
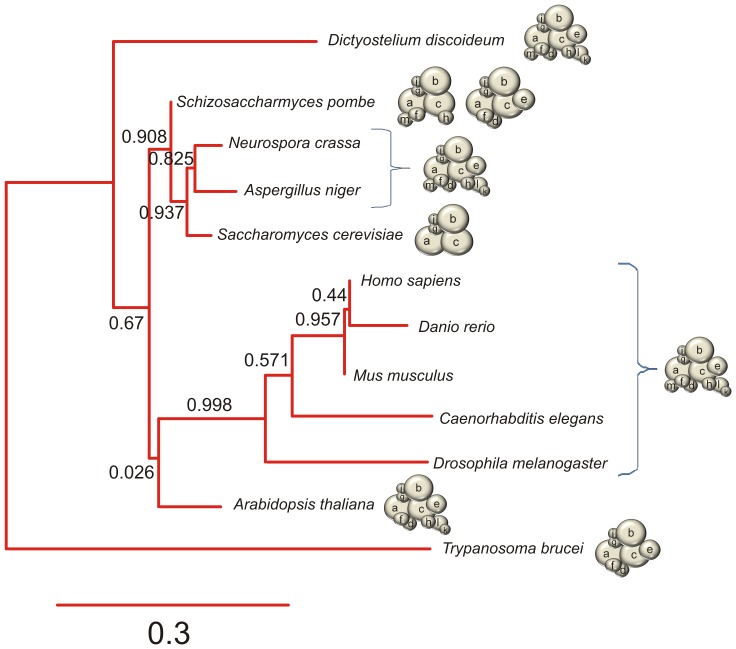
The stoichiometric subunit composition of eIF3 varies across species. Cladogram constructed using sequences of 18S rRNA from the listed organisms. The subunit composition of eIF3 from each organism is depicted using spherical models. Subunit count in the stoichiometric complex of the displayed organisms is as follows: *H. sapiens, D. rerio, M. musculus, C. elegans, D. melanogaster, N. crassa, D. discoideum and A. niger* (12); *A. thaliana* (11); *S. pombe* (Csn7b, left, or Int6, right, complexes) (8 each); *T. brucei* (8); *S. cerevisiae* (5), The size of the spheres used to depict eIF3 subunits are relative to their respective molecular weights. The tree was constructed using tools at www.phylogeny.fr [46].

The subunit composition of eIF3 varies dramatically among organisms, typically with eIF3 complexes missing subunits as species diverge from metazoa (Figure 1). Most genetic and biochemical studies of eIF3 have been performed with the yeast *Saccharomyces cerevisiae*, which contains only five stoichiometric subunits (eIF3 a, b, c, g and i) and the fission yeast *Schizosaccharomyces pombe* which contains two distinct, eight subunit complexes (eIF3 a, b, c, f, g, h, i, m or eIF3 a, b, c, d, e, f, g, i) [2]. The five subunits from *S. cerevisiae* eIF3 have been proposed to comprise the core of eIF3 in all eukaryotes [2]. However, the minimal stable core structure of human eIF3 is composed of eight subunits (a, c, e, f, h, k, l and m), only two of which are conserved with the *S. cerevisiae* eIF3 complex [12], [13]. Thus, a genetically tractable model system with an eIF3 that more closely corresponds to that in humans would greatly aid studies of the assembly and function of this essential translation factor.

## Results and Discussion

### Essential and Non-essential Subunits in *Neurospora* eIF3

The filamentous fungus *Neurospora crassa* (Nc) is a morphologically complex, multicellular, model organism with at least 28 distinct cell-types [14]. The sequenced *Neurospora crassa* genome contains annotated orthologues of 10 eIF3 subunits, with eIF3j, k, and m remaining unannotated [15]. We conducted BLASTp searches of the *Neurospora* genome using human eIF3 query sequences and identified orthologues of eIF3k and m [16]. Using an eIF3j query from *Aspergillus niger,* we also identified a orthologue for eIF3j. Reciprocal BLASTp searches against the human database using *Neurospora* eIF3 subunit queries corroborated all 13 eIF3 subunit orthologues (Table 1) making *Neurospora* an attractive model system for studying human-like eIF3.

**Table 1:** *N. crassa* eIF3 genes, identity to their human orthologues and knock-out phenotypes.

eIF3 subunit	*N. crassa* gene (NCU#)	UniProt Accession Number		% identity	KO phenotype
		*N. crassa*	Human		
a	00040	Q7RWT1	Q14152	37	lethal
b	02208	Q7S464	P55884	39	lethal
c	07831	Q7SBD4	Q99613	39	lethal
d	07380	Q7S212	O15371	39	lethal
e	05889	Q7S519	P60228	43	viable
f	01021	Q9P748	O00303	32	lethal
g	08046	Q6MFP4	O75821	38	lethal
h	07929	Q7S9Y9	O15372	30	viable
i	03876	Q7RXH4	Q13347	52	lethal
j	07954	Q7S931	O75822	24	viable
k	09707	Q7S2R9	Q9UBQ5	29	viable
l	06279	Q7SB62	Q97262	51	viable
m	02813	Q7SEK1	Q7L2H7	26	lethal

To assess the importance of eIF3 subunits to *Neurospora* viability, *Neurospora* knock-out strains were first propagated asexually. Knock-outs that are null viable can be isolated as homokaryons, in which the strain only contains nuclei with the gene of interest deleted. Alternatively, knock-outs that are null lethal cannot be isolated as homokaryons, but instead can be maintained as heterokayons in which nuclei from a compatible strain complement the null lethal phenotype through hyphal fusion [17]. Knock-out strains of 12 *Neurospora* eIF3 subunit orthologues were obtained from the Fungal Genetics Stock Center (FGSC) [18] (Table S1 in File S1), all of which have the knocked out gene replaced with a hygromycin B resistance gene [19]. Knock-outs of subunits e, h, j, k and l were confirmed to be homokaryons by PCR genotyping, Southern blotting and the ability to cross *his-3* genotypes into each strain. Knock-outs of eIF3a, c, d, f, g, i and m were obtained from the FGSC as heterokaryons, implying these knock-outs are null lethal.

To verify the lethality of deleting subunits eIF3a, c, d, f, g, i or m, we crossed each knock-out strain with wild-type *Neurospora* and re-selected strains in the presence of hygromycin B, the marker used in the original knock-out cassettes. Ascospores plated on media with hygromycin B failed to grow, suggesting that the knock-outs were either null lethal, or could not germinate in the presence of hygromycin. To test the latter possibility, ascospores from the same cross were germinated on media that did not contain hygromycin to yield growing progeny. Conidia from these progeny were subsequently transferred to media containing hygromycin B, but failed to grow in the presence of hygromycin B, indicating the germinated progeny did not contain knock-out cassettes. Furthermore, PCR genotyping of conidia of germinated ascospores indicated the presence of only the wild-type loci. Taken together, these data indicate that subunits eIF3a, c, d, f, g, i and m are essential, whereas subunits eIF3e, h, j, k and l are dispensible for eIF3 function in *N. crassa* (Table 1).

A knock-out of subunit eIF3b in *Neurospora* could not be obtained even in the form of a heterokaryon. Based on phylogenetics (Figure 1), deletion of subunit eIF3b is expected to be null lethal [20]. To test whether eIF3b is essential, we used the mechanisms of meiotic silencing of unpaired DNA (MSUD) and repeat induced point mutation (RIP) in *Neurospora.* The MSUD mechanism silences all copies of duplicate genes during meiosis, including homologous genes that are paired [21]. RIP recognizes and mutates duplicate genes during pre-meiotic stage of the sexual cycle [22]. We inserted a duplicate gene of eIF3b at the *his-3* locus and subsequently crossed the homokaryon with wild-type *Neurospora*. The unpaired or duplicate eIF3b gene would be recognized by MSUD or RIP machinery, respectively. If eIF3b is essential before or during meiosis we would expect a barren phenotype or few viable ascospores produced from the cross [21], [22]. The cross resulted in a barren phenotype, in which normal-looking perithecia shoot few or no ascospores. Thus, eIF3b is also an essential subunit in *N. crassa* (Table 1).

### Phenotypes of Strains with Deletions of Nonessential eIF3 Subunits

**Figure 2:**
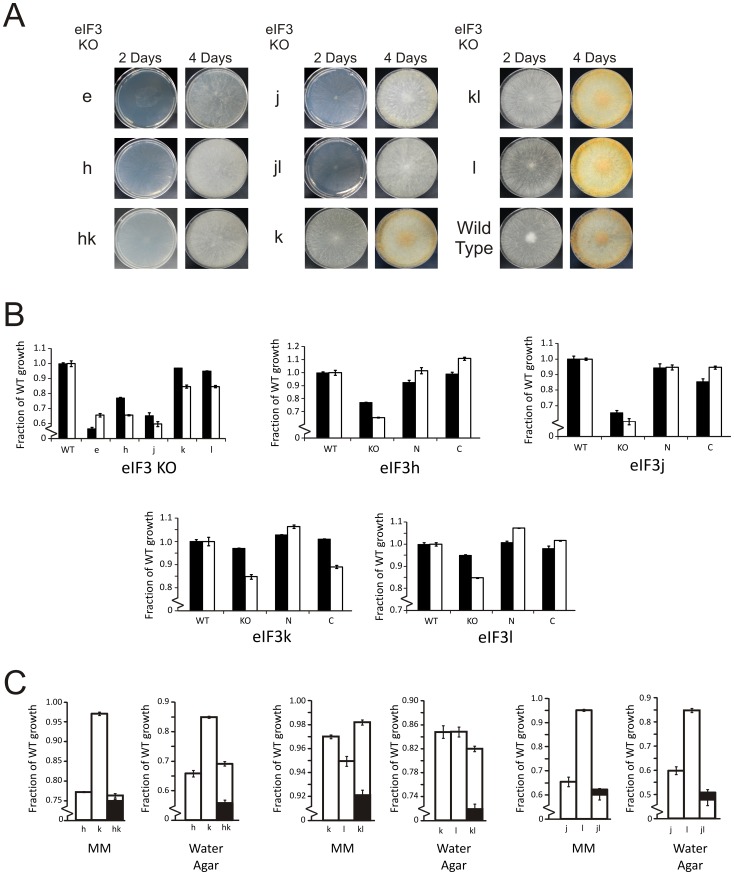
Viable Nc eIF3 knock-out strains display defects in conidiation and linear growth. (A) Growth of Nc eIF3 single and double knock-out (KO) strains on Vogel’s minimal media (MM) with 2% sucrose. Plates were spotted from frozen stocks of the indicated eIF3 subunit single or double knock-out strain, grown in the dark and photographed on the indicated days. (B) Linear growth of Nc eIF3 knock-out strains on Vogel’s MM with 2% sucrose (black bars) or water agar (white bars). In all graphs linear growth is plotted as a fraction of wild-type growth on comparable media. Linear growth of wild-type (WT) on water agar as a fraction of MM with 2% sucrose is 0.63 (Table S2 in File S1). Linear growth of eIF3 single knock-out strains is compared to WT, where the eIF3 subunit knock-out is indicated below the bars (graph eIF3 KO). Linear growth of eIF3 knock-out compared to wild-type and the knock-out strain with an N-terminally tagged (N) or C-terminally tagged (C) recombinant eIF3 subunit at the *his-3* locus (graphs eIF3h, j, k, l). The recombinant eIF3 subunit and corresponding KO strain is indicated below each graph. (C) Linear growth of eIF3 double knock-out strains compared to the corresponding single knock-outs (white bars) on Vogel’s MM with 2% sucrose and 2% agar or Vogel’s MM with 2% agar (water agar). Black bars represent the calculated multiplicative effect from both single knock-outs. Each graph has a broken vertical axis to visually emphasize the difference in linear growth rates. Error bars indicate the standard error on the mean. Numerical values for linear growth and errors are in Table S2 in File S1.

*Neurospora* knock-out strains for the nonessential subunits eIF3e, h, j, k and l were analyzed for the phenotypic effect of subunit deletion. The ΔeIF3e strain was aconidial, whereas the ΔeIF3j and ΔeIF3h strains had noticeable conidiation defects (Figure 2A). The ΔeIF3h strain also had reduced aerial hyphae (Figure 2A, Figure S1 in File S1). By contrast, the ΔeIF3k and ΔeIF3l strains appeared to grow and conidiate similarly to wild-type (Figure 2A). To more quantitatively assess the effects of eIF3 subunit deletion, we compared the linear growth rates of eIF3 subunit knock-out strains to wild-type *N. crassa* in race tubes. On sucrose minimal media, the ΔeIF3e strain exhibited the most severe linear growth phenotype (Figure 2B, Table S2 in File S1). Knock-outs of eIF3h or eIF3j also greatly reduced linear growth (Figure 2B, Table S2 in File S1). Although the effects were small by comparison, deletion of eIF3k or l also resulted in measurable and reproducible reductions in linear growth (Figure 2B, Table S2 in File S1). To test whether cellular stress might increase the severity of eIF3 knock-out phenotypes compared to the wild-type strain, we tested linear growth of *Neurospora* on water agar (See Materials and Methods). As anticipated, linear growth on water agar exacerbated the eIF3 knock-out phenotypes (Figure 2B, Table S2 in File S1), except for the ΔeIF3e strain, which grew better on water agar relative to wild-type *Neurospora* (Figure 2B, Table S2 in File S1). Taken together, these data reveal that many non-universally conserved subunits of *Neurospora* eIF3 are essential in human-like eIF3 complexes, whereas deletion of some subunits result in *Neurospora* eIF3 sub-complexes that maintain much of its biological function.

To further probe eIF3 subunit dispensability and genetic interactions, we constructed three double knock-out strains of Nc eIF3: deletion of subunits k and l (ΔeIF3kl), deletion of subunits h and k (ΔeIF3hk) and deletion of subunits j and l (ΔeIF3jl). We compared the linear growth of these double knock-out strains to each individual deletion, as well as to the predicted multiplicative effect of combining deletions (Figure 2C, Table S2 in File S1). Linear growth rates on sucrose minimal media or water agar were greater than predicted for both the ΔeIF3kl and ΔeIF3hk strains (Figure 2C, Table S2 in File S1), indicating synergistic epistatic effects. The ΔeIF3jl strain had linear growth rates similar to predicted rates (Figure 2C, Table S2 in File S1), indicating epistatic neutrality. Linear growth rates on water agar were also much greater than the predicted values for the ΔeIF3kl and ΔeIF3hk strains, indicating strong compensatory effects for the double knock-out (Figure 2C, Table S2 in File S1). These results show that the subunit pair of eIF3k and eIF3l and the subunit pair of eIF3h and eIF3k have structural or genetic synergy.

### Biochemical Properties of eIF3 and eIF3j Isolated from *Neurospora crassa*

**Figure 3:**
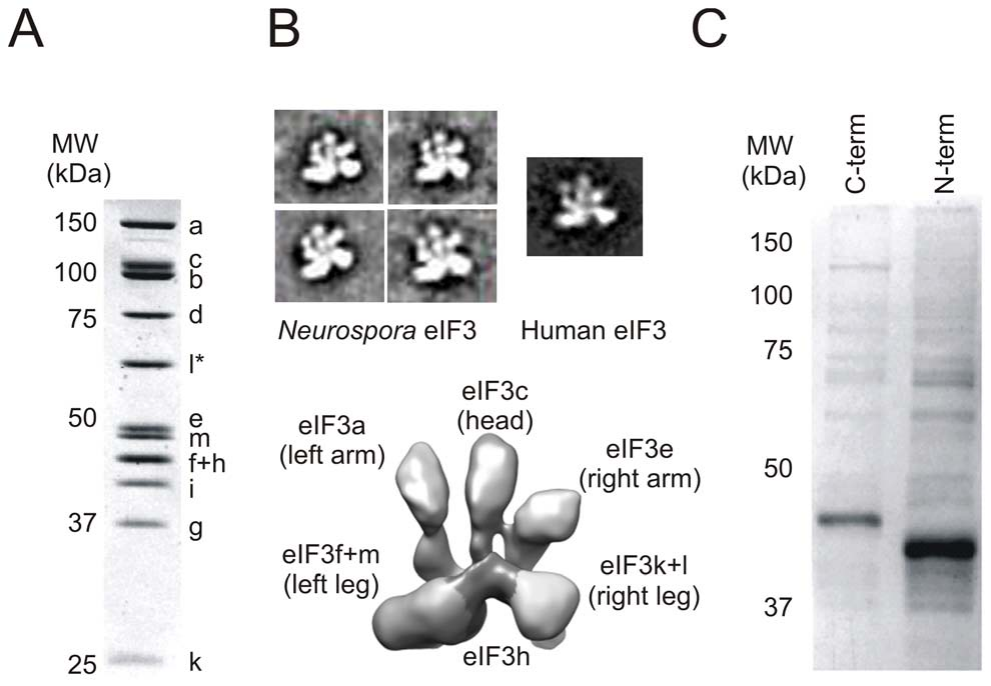
Composition and structure of eIF3 assemblies. (A) Coomassie stained purified *N. crassa* eIF3 with C-terminally tagged eIF3l. Subunits identified by tandem mass spectrometry are labelled. Subunits f and h co-migrate on the gel. The tagged subunit is indicated with an asterisk. (B) 2-D EM class averages of negatively stained *N. crassa* eIF3 dodecamer compared with the equivalent view of the human eIF3 octamer. The 3-D cryo-EM reconstruction of the human eIF3 octameric core is shown for reference, with labeled features and subunits (adapted from [11]). (C) Coomassie stained affinity purifications of FLAG tagged *Neurospora* eIF3j (C- or N-terminally tagged). The major bands are Nc eIF3j. Minor bands are interacting proteins (Table S3 in File S1).

**Figure 4:**
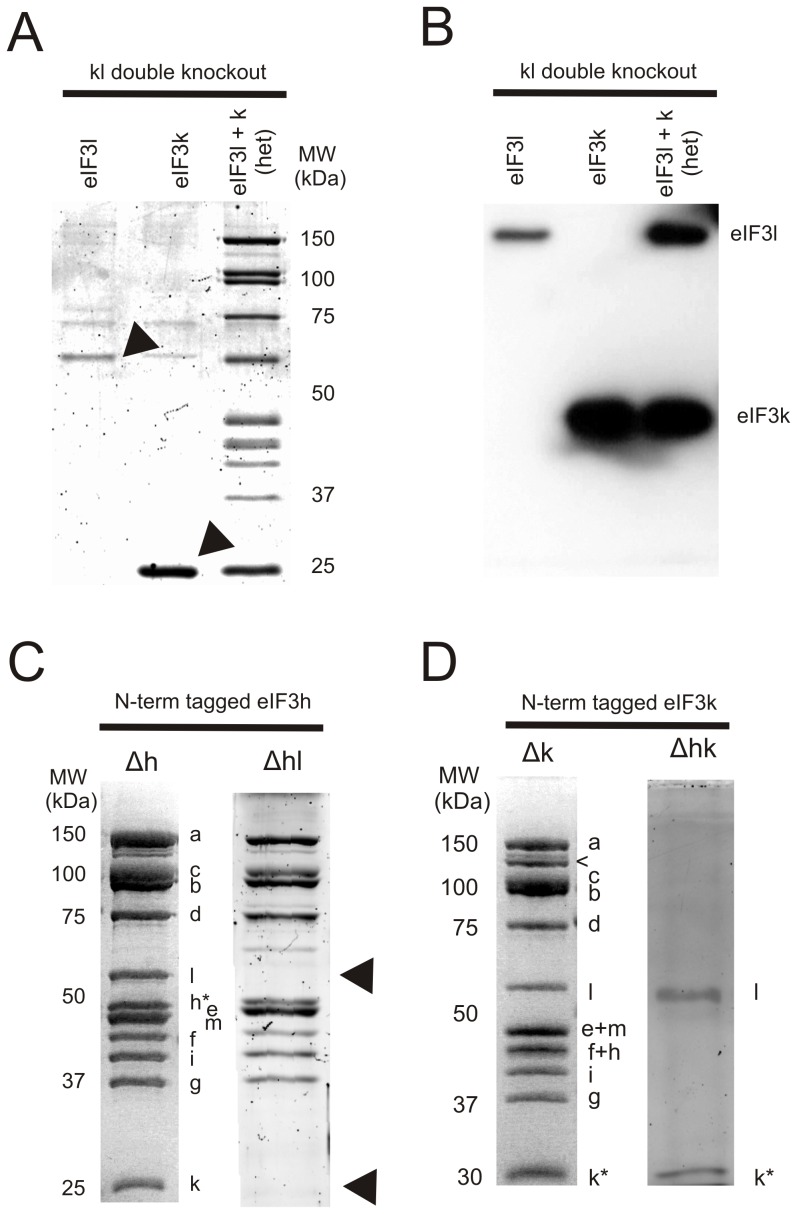
Affinity purification of eIF3 knock-out strains reveal subunit interdependence during eIF3 assembly. (A) Anti-FLAG affinity purifications from *N. crassa* extracts using N-terminally tagged eIF3l or eIF3k in the ΔeIF3kl double knock-out background (lanes 1 and 2). Affinity purification from *N. crassa* extract obtained from a heterokaryon strain (lane 3) made from strains used in lanes 1 and 2. N-terminally tagged k and l subunits expressed from the *his-3* locus are indicated above each lane. All strains are ΔeIF3kl double knock-outs. Contaminant bands in lanes 1 and 2 were identified as phenylalanyl tRNA synthetase beta chain (73 kDa) and tRNA ligase (58 kDa). (B) Anti-FLAG Western blot of the affinity purifications from the gel in (A). Each lane has the exact amount of total protein loaded in (A). Anti-FLAG affinity purifications from *N. crassa* extracts using N-terminally tagged eIF3h in ΔeIF3h or ΔeIF3hl strains (C) or N-terminally tagged eIF3k in ΔeIF3k or ΔeIF3hk strains (D). The tagged h or k subunits are indicated with asterisks. Arrows indicate the missing k and l subunits in the ΔeIF3hl strain (C) or a degradation product of eIF3a (D). All gels in panels (A), (C) and (D) are stained with Coomassie blue.

To determine the structural integrity of the Nc eIF3 complex, subunits eIF3h, j, k or l were individually inserted with N- or C-terminal affinity tags into their respective knock-out background strains, at the *his-3* locus (Table S1 in File S1). Each tagged subunit rescued most or all of the linear growth phenotype observed with the respective knock-out strain (Figure 2B), with the exception of the C-terminally tagged eIF3k on water agar, indicating the recombinant constructs were functional *in vivo*. Using strains with tagged eIF3h, k or l, we isolated eIF3 complexes with 12 stoichiometric subunits (Figs. 3A, 4C and D), all corresponding to orthologues of human eIF3 subunits (a-m, excluding j). Analysis of this stoichiometric dodecamer by negative stain electron microscopy revealed that it has the same anthropomorphic features as human eIF3 (Figure 3B) [12], [13]. Thus, like human eIF3, *Neurospora* eIF3 contains 12 stoichiometric subunits and is the first structurally human-like eIF3 to be purified and visualized outside of metazoa.

Notably, Nc eIF3j was not present in any of the purified eIF3 samples (Figs. 3A, 4C and D), similar to the reported behavior for *S. cerevisiae* eIF3j (Hcr1p) [23]. However, tagged Nc eIF3j was able to affinity purify a myriad of translation-related proteins including eIF3 subunits, ribosomal proteins and other translation initiation/elongation factors (Figure 3C and Table S3 in File S1), also as seen with *S. cerevisiae* [24] and *S. pombe* [25]. Taken together, these results suggest that *Neurospora* eIF3j, like its yeast counterparts, associates with the eIF3 complex in a weak manner. Together with the neutral epistasis seen with the ΔeIF3jl strain, these data indicate that Nc eIF3j functions in translation in *Neurospora crassa*.

### Assembly of eIF3k and l into eIF3 Complexes

Reconstitution of human eIF3 revealed that eIF3h, k and l are all required for the formation of a stable eight-subunit core composed of the subunits containing Proteasome Cop-9 Initiation factor 3 (PCI) or Mpr1-Pad1 N-terminal (MPN) domains [12]. Consistent with human eIF3 reconstitutions, the ΔeIF3h strain exhibited appreciable defects in linear growth and developmental phenotypes (Figure 2A and B, Table S2 and Figure S1 in File S1). Surprisingly, the *Neurospora* ΔeIF3kl strain exhibited nearly wild-type linear growth rates and conidiation on sucrose minimal media (Figure 2A and B, Table S2 in File S1). These results suggest that subunits k and l are not required for assembly of the remainder of the eIF3 complex *in vivo*.

To better understand the role of eIF3k and l in eIF3 assembly, we used the ΔeIF3kl strain, and inserted either N-terminally tagged eIF3k (strain ΔeIF3kl, +N-k) or eIF3l (strain ΔeIF3kl, +N-l) at the *his-3* locus to assess the stability of the eIF3 complex in the absence of l or k, respectively (Table S1 in File S1). Neither N-terminally tagged subunit was able to affinity purify the remainder of the eIF3 complex (Figure 4A and B). Furthermore the level of tagged eIF3l in the ΔeIF3kl background was much lower compared to tagged k in the ΔeIF3kl background (Figure 4B), suggesting that subunit eIF3l is less stable in the absence of eIF3k (Figure S2 in File S1). Remarkably, mixing of lysates from the (ΔeIF3kl, +N-k) and (ΔeIF3kl, +N-l) strains–which contain free eIF3k and eIF3l–did not result in their assembly into the remaining 10-subunit complex (Figure S3 in File S1). However, the entire dodecameric complex could be affinity purified from a heterokaryon strain generated from the above two single-deletion strains (Figure 4A). These results indicate that subunits eIF3k and eIF3l require each other, as well as additional *in vivo* factors, to be incorporated into the dodecameric eIF3 complex.

Notably, a third strain containing a tagged h subunit in a ΔeIF3hl background (strain ΔeIF3hl, +N-h) yielded affinity-purified complexes lacking both eIF3k and l subunits (Figure 4C). Affinity purification of N-terminally tagged eIF3k in the absence of eIF3h (strain ΔeIF3hk, +N-k) yielded an eIF3kl dimer (Figure 4D) indicating that eIF3k and l are either pre-assembled as a dimer and interact with eIF3 already containing the h subunit or alternatively the eIF3kl dimer binds to eIF3h and assembles with eIF3 as a trimer. These data corroborate the knock-out phenotypes, in which a ΔeIF3hk double deletion strain has essentially the same linear growth fitness as the ΔeIF3h single deletion (Figure 2C, Table S2 in File S1), i.e. both strains are functionally triple deletion strains. Taken together these data show that assembly eIF3h into eIF3 occurs in the absence of eIF3k and eIF3l. Furthermore, the assembly of eIF3k and eIF3l into eIF3 is dependent on eIF3k-eIF3l interactions as well as on eIF3h.

### The Right Side of eIF3 is Dispensable *in vivo*

When compared to the subunit positions in the structural core of human eIF3 [11], the eIF3 subunits that are dispensable in *N. crassa* (e, h, k and l) are localized to the right arm and right leg of eIF3 (Figure 3B) [12]. Strikingly, our data show that at least three subunits (eIF3h, k and l; ΔeIF3hk strain) can be removed from the complex with the resulting strain remaining viable (Figures 2C and 4C), analogous to one of the two eIF3 complexes in *S. pombe* (Figure 1). Based on the recent cryo-EM structure of a mammalian PIC-like complex, eIF3 binds the platform of the human 40S ribosomal subunit with the right arm, left and right legs positioned away from the ribosomal subunit interface [3]. Thus, subunits eIF3e, h, k, l and m–positioned away from the 40S subunit, may regulate translation initiation by serving as a binding platform for translation or regulatory factors. Consistent with this model, the predominant isoform of eIF3h in zebrafish regulates the translation of proteins involved in development [8]. A similar role for eIF3h has also been observed in *Arabidopsis* [26], [27], [28] and *S. pombe* [29]. In *N. crassa*, deletion of eIF3h also results in developmental defects in conidiation (Figure S1 in File S1, Figure 2A). Thus, the links between eIF3h and development are conserved among species spanning a large swath of eukaryotic phylogeny. Importantly, our results indicate that defects in eIF3h likely impact the function of subunits eIF3k and eIF3l as well. Future experiments will be required to determine how subunits eIF3h, k and l work together to regulate the translation of specific mRNAs.

### Neurospora eIF3 Reconciles a Model for Human-like eIF3 Assembly

All four dispensable subunits, including subunits in double knock-outs (ΔeIF3hk and ΔeIF3kl) of the *Neurospora* dodecameric complex, were essential for the stable formation of the reconstituted human octameric core (subunits a, c, e, f, h, k, l and m) in *Escherichia coli* [12]. Similarly, reconstitution of functional human eIF3 in HeLa *in vitro* translation extracts or insect cells required subunits e and h [30], [31]. By contrast, the viability of *Neurospora* eIF3e, h, k and l knock-out strains suggest that other subunits not present in the PCI-MPN core (e.g. subunits b, c, d, g, i) or molecular chaperones might contribute to the *in vivo* stability of eIF3 compared to the reconstituted systems. The co-dependence of eIF3k and eIF3l assembly into eIF3 provide the first direct evidence for subunit interdependence in endogenous eIF3 assembly, and also suggest that cellular factors may contribute to the process. Notably, the co-dependence of k and l is phylogenetically supported by the fact that k and l are present or absent in a pair-wise manner in other eukaryotic genomes (Figure 1 and [2], [32]).

**Figure 5:**
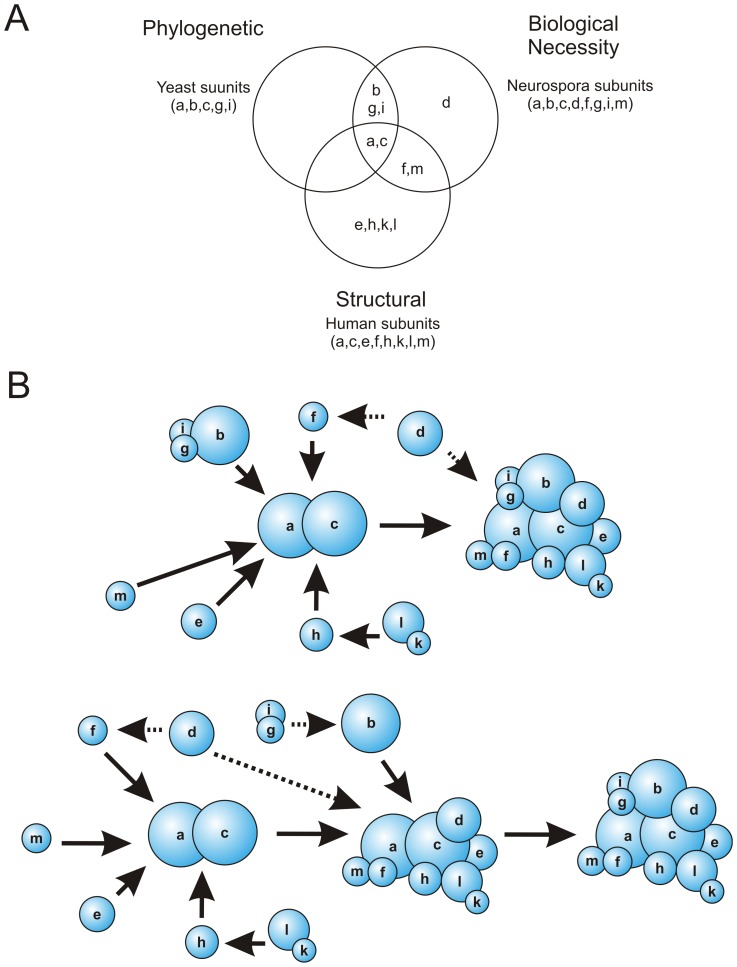
Redefining the core of eIF3 and proposed models for assembly. (A) Venn diagram highlighting common subunits among three definitions of the eIF3 core complex: 1) the phylogenetically conserved complex from *S. cerevisiae*, 2) the reconstituted human PCI-MPN core complex and 3) the biologically essential subunits from *N. crassa* eIF3. (B) Proposed models for human-like eIF3 assembly. Individual subunits or sub-complexes assemble onto an ac dimer. Solid and dashed arrows represent assembly and alternative assembly interactions and are drawn to reconcile the compositional breadth of eIF3 sub-complexes across species as well as biochemical data [12], [47].

Models that define the core of eIF3 differ depending on whether they rely on minimal subunit composition using phylogenetics (Figure 1) [2], on cell viability (Table 1), or on structural information [12], [23], [30], [33], [34]. By comparing all three models of the core of eIF3 (Figure 5A), we propose that eIF3 assembles on a dimer composed of subunits a and c (Figure 5A). The remaining subunits would then be assembled onto the eIF3a/eIF3c (ac) dimer either as single subunits or sub-complexes (Figure 5B). In support of this assembly model, we recently demonstrated the ac dimer to be a minimal eIF3 sub-complex capable of high affinity binding to the hepatitis C virus internal ribosome entry site (HCV-IRES) [35]. Our phenotypic analyses of eIF3e, h, k or l knock-outs suggest that these subunits likely assemble onto the ac dimer [12] in an ordered manner (Figure 4), but independently of the remaining essential subunits (b, d, f, g, i and m) (Figure 5B). In particular, subunits eIF3k and l are assembled into eIF3 as a dimer (Figure 4D), dependent on the incorporation of eIF3h into eIF3 (Figure 4C). Ordered assembly of subunits onto the ac dimer could allow the levels of eIF3 to be regulated by simply regulating the expression of subunits a and c, as observed in human cells [36].

### Conclusion

We have used the power of *N. crassa* genetics and biochemistry eIF3 to map the structure and function of its compositionally human-like eIF3 translation initiation factor *in vivo*. Four subunits within the stable dodecameric complex (e, h, k and l) and the eIF3j orthologue are dispensable for growth, and three double knock-out strains (ΔeIF3kl, ΔeIF3hk and ΔeIF3jl) are also viable. We also show the strong interdependence of eIF3h, k and l assembly into eIF3, which has important implications for the mechanism by which eIF3h regulates translation of specific mRNAs in animals and plants [8], [26], [27]. When compared with phylogenetic and reconstitution data, the results with *N. crassa* eIF3 allow us to redefine the core of eIF3 as the eIF3a/eIF3c dimer, onto which individual subunits or eIF3 subcomplexes assemble, possibly with the aid of *in vivo* factors. *N. crassa* eIF3 should therefore serve as a useful genetic and biochemical system for unravelling the assembly of human-like eIF3 and its roles in regulating translation in living cells.

## Materials and Methods

### Creation of *Neurospora crassa* eIF3 Expression Constructs

All eIF3 cDNAs were prepared from wild-type *Neurospora* total RNA (Trizol extracted) using the M-MLV reverse transcriptase (Invitrogen) with poly-dT reverse primers (first strand) and subsequent PCR amplification with eIF3 gene specific primers (second strand). PCR was used to introduce in-frame PreScission protease cleavage sites either upstream (N–terminally tagged) or downstream (C-terminally tagged) of the eIF3 gene, allowing the option of removing the affinity tag by proteolytic cleavage with PreScission protease. All constructs were cloned into the *Asc*I and *Pac*I restriction sites of plasmid pCCG::N-FLAG::HAT (N-terminally tagged; FJ457007) or into the *Xba*I and *Pac*I restriction sites of plasmid pCCG::C-Gly::HAT::FLAG (C-terminally tagged; FJ457003) [37]. Plasmids were obtained from the FGSC [18]. Both constructs add FLAG and HAT affinity tags, separated by glycine linkers, to the terminus of the eIF3 subunit. The N-terminal affinity tags chosen were FLAG-3xGly-HAT-5xGly, and the C-terminal affinity tags were 10xGly-HAT-5xGly-FLAG. The plasmid constructs were designed to target the *his-3* locus and expression was controlled by the constitutive promoter CCG-1. Plasmid constructs were transformed and selected using *Escherichia coli*.

### *N. crassa* Strains

All strains used in this study are summarized in Table S1 in File S1. Knock-out strains, obtained from the Fungal Genetics Stock Center (FGSC) are indicated in Table S1 in File S1 [18]. *His-3* auxotrophs of eIF3h, j, k or l were created by crossing with the *his-3* strain of the opposite mating type and selecting hygromycin resistant progeny on Vogel’s minimal media, 2% agar, 2% sucrose supplemented with 100 µg/mL histidine and 200 µg/mL hygromycin B. Strains isolated from the cross that were not histidine auxotrophs were used in phenotypic analyses (described below), whereas auxotrophic strains were used to insert tagged eIF3 subunits. Strains expressing N- or C-terminally tagged eIF3 constructs (subunits h, j, k or l) were generated by electroporating 10 µL of 100 ng/µL plasmid DNA of the tagged eIF3 construct into 90 µL of conidia (∼1×10^9^ conidia/mL in 1 M ice cold sorbitol) from their respective *his-3* eIF3 knock-out strains to recreate the wild-type complex containing an affinity tag. Transformants were purified by back crossing with the *his-3* strain and selecting on Vogel’s minimal media, 2% agar, 2% sucrose supplemented with 200 µg/mL hygromycin B or by purifying microconidia using a protocol modified from http://www.fgsc.net/fgn37/ebbole1.html. Microconidia purification was done by plating a small amount of conidia from the transformant onto water agar (Vogel’s minimal media, 2% agar) and growing at 30°C in the dark for 4 days, followed by at least one week at room temperature in the light. Microconidia were harvested by scraping the water agar plates with 2 mL water, filtering the collected water through a 5 µm filter to remove macroconidia. Microconidia were pelleted by centrifugation, resuspended in ∼50–100 µL of water and plated on Vogel’s minimal media, 2% agar, 2% sucrose plates. Plated microconidia were germinated for 15–30 hours at 30°C, picked under a dissecting microscope and transferred to individual slants containing Vogel’s minimal media, 2% agar, 2% sucrose.

Double knock-out strains were created by crossing single knock-outs of opposite mating type. Double knock-outs that contained tagged eIF3 subunits at the *his-3* locus were either made by crossing one knock-out with another strain that contained the other knock-out and tagged subunit or by creating a *his-3* auxotroph of the double knock-out and transforming in the tagged subunit as described above.

### *N. crassa* Strain Validation

Neurospora genotypes were assessed first using PCR with primer pairs that included either a reverse primer that binds in the hygromycin cassette, which is inserted in the reverse orientation, along with a forward primer in the in the 5′ flanking region (hyg+5′) or a forward primer that binds in the hygromycin cassette along with a reverse primer in the in the 3′ flanking region (hyg+3′). These primer pairs provided a quick way of identifying whether or not the correct knock-out cassette was present. We further assessed strain purity (homokaryon vs. heterokaryon) by Southern blotting. Probes were made using the DIG Probe synthesis kit (Roche). Probes were made using the same primer pairs used for PCR identification and thus contained a flanking region and the complete hygromycin cassette. This enabled the identification of both wild-type and knock-out cassettes using a single probe. Genomic DNA (gDNA) preparations were digested overnight with restriction enzymes to test for specific knock-out cassettes (KO/enzyme/probe): (eIF3e/*Eco*RI/hyg+5′ and hyg+3′), (eIF3h/*Xho*I/hyg+5′), (eIF3j/*Xho*I/hyg+5′ and hyg+3′), (eIF3k/*Sma*I/hyg+3′), (eIF3l/*Eco*RI/hyg+3′) and resolved on 0.8% agarose gels overnight at ∼17 mA. In all cases wild-type gDNAs were analyzed in parallel. Blotting was done using the Easy Hyb system (Roche). Tagged eIF3 genes at the *his-3* locus were identified by PCR using forward and reverse primers that bind to the N-terminal or C-terminal tags, respectively, along with a gene-specific reverse primer. Expression of the protein was confirmed by Western blot analysis of whole cell extracts using anti-FLAG monoclonal antibody HRP conjugate (Sigma).

### Phenotype Assays

Lethal eIF3 knock-out strains were validated by crossing heterokaryons obtained from the FGSC (see Table 1) with wild-type *Neurospora*. Reciprocal crosses were done using the wild-type strain as either the male or female. Ascospores from each cross were harvested and germinated on Vogel’s minimal media, 2% agar, 2% sucrose supplemented with or without 200 µg/mL hygromycin B. No ascospores germinated on media containing hygromycin. Conidia harvested from germinated ascospores grown on non-hygromycin media subsequently failed to germinate when transferred to slants that contained Vogel’s minimal media, 2% agar, 2% sucrose supplemented with 200 µg/mL hygromycin B.

Growth and conidiation phenotypes were assessed by spotting a small amount of frozen conidia in the middle of a plate containing Vogel’s minimal media, 2% agar and 2% sucrose. Plates were incubated at 30°C in the dark and photographed at the indicated times in Figure 2.

Race tubes were made using 25 mL serological pipettes. Autoclaved media (Sucrose media: Vogel’s minimal media, 2% agar, 2% sucrose or water agar: Vogel’s minimal media, 2% agar) was drawn up to the top of the pipette and expelled to the 13 mL line, at which point the pipette was laid down on a level surface until the media solidified. The tips of the pipettes were broken off and capped with a sterile cap right before spotting. Race tubes were spotted with conidia harvested using water from slants that had been grown at 30°C in the dark for 2–3 days and allowed to conidiate in the light for an additional 7–8 days. All strains within an independent race tube experiment used conidia of equal age. All race tube data were collected and analyzed in parallel with wild-type samples each time. After spotting, the race tubes were incubated at 30°C in the dark for 16–18 hours before taking the initial measurement. Subsequent measurements were typically taken in 6–8 hour increments over the course of 2–3 days. Linear growth was normalized to the first time point. Three technical replicates of each biological replicate were combined and plotted against time (hours) and fit with a linear function using Sigmaplot. The linear growth rate (slope) and errors on the slope of the linear fits were normalized as a fraction of the linear growth of wild-type. The presented data represent the combination of at least two independently isolated isogenic biological replicates, each with a minimum of three technical replicates. Knock-out strains transformed with empty vectors, which express only the HAT-FLAG affinity tag, had the same linear growth as their respective untransformed knock-out strain.

For double knock-out and epistatic analysis, the predicted double knock-out linear growth phenotypes were calculated by multiplying the relative linear growth rates of the individual knock-outs. Predicted double knock-out errors were calculated using the root of the sum of squares of the standard errors of the individual knock-outs.

### Isolation of eIF3 Complexes or eIF3j from *N. crassa* Lysates

Strains containing tagged eIF3 subunits were grown in liquid culture (Vogel’s minimal media, 2% sucrose) for 40–46 hours from an initial conidial density of OD_600_ = 0.04. Mycelia were collected using a Büchner funnel with filter paper, rinsed briefly with deionized water and immediately transferred to dry ice. Frozen biomass was ground up using a mortar and pestle in liquid nitrogen. Lysis buffer (TBS, 0.5% Triton X-100, 10% glycerol and protease inhibitors (Roche) was added directly to the powered biomass (∼10–15 mL lysis buffer per gram of biomass) and further ground with the mortar and pestle until the lysate was an even consistency. The lysate was cleared by centrifugation and filtered with a 0.2 µm syringe filter. Anti-FLAG affinity beads (Sigma) were added directly to the lysate for 4–20 hours at 4°C, collected by centrifugation and washed 5 times each with ∼10 bed volumes of wash buffer (TBS, 10% glycerol). The complex was eluted using FLAG peptide (Sigma). The eluate was washed three times with ∼10 volumes of wash buffer containing 1 mM DTT though an Amicon Ultra 0.5 mL Ultracel 30k centrifugal filter to remove the FLAG peptide.

### Analysis of eIF3j Interacting Proteins by LC MS/MS

*N. crassa* strains containing N- or C-terminally tagged eIF3j were grown and eIF3j and its interacting proteins were purified as indicated above. Strains that expressed only the affinity tag at the *his-3* locus were used as negative controls to determine *Neurospora* proteins that bind non-specifically to the anti-FLAG affinity resin. Total eluates (∼5–25 µg of total protein from eIF3j eluates, negative controls typically yielded <1 µg of total protein) were denatured, reduced and acetylated before digesting with trypsin. Trypsin digestions were desalted as previously described [25] and used for LC MS/MS analysis as previously described [38]. Table S3 in File S1 lists interacting proteins that were identified in the eluates from four replicates (two each with N- and C-terminally tagged eIF3j). Proteins listed in Table S3 in File S1 occurred in at least two of the four replicates and a minimum of four peptides total. Proteins identified from negative controls were excluded in Table S3 in File S1.

### Electron Microscopy

Protein samples were diluted to a final concentration of 50 nM in buffer 20 mM Hepes, pH = 7.4, 120 mM KCl, 0.5 mM EDTA, 1 mM DTT, and 3% trehalose. 400 mesh continuous carbon grids were plasma cleaned in a 75% Ar/25% O_2_ atmosphere for 20 seconds in a Solarus plasma cleaner (Gatan, Inc). Sample aliquots of ∼4 µL were placed onto the grids, negatively stained with a 2% uranyl acetate solution and blotted to dryness. Data were acquired using a Tecnai T12 electron microscope operating at 120 keV using the Leginon automated data collection software [39] on a Tietz 4×4K pixel CCD camera (15 µm pixel size) at a nominal magnification of 50,000X (2.18 Å/pixel). Images were collected in low-dose mode with a dose of ∼20 e-/Å^2^ and a defocus range from −0.5 to −1.5 µm.Two-dimensional data processing was performed using programs and utilities contained within the Appion processing environment [40]. Particles were initially extracted using a difference of Gaussians (DoG) particle picker [41]. After contrast transfer function (CTF) estimation using CTFFind [42], particle image stacks were generated by extracting selected particles with a box size of 192×192 using the “batchboxer” program [43] with an estimated CTF confidence cutoff above 80%. The data were then binned by a factor of two for processing. Each particle was normalized using the XMIPP normalization program [44] using a cutoff of 4.5σ of the mean pixel value. The stack was subjected to several rounds of reference-free two-dimensional classification using IMAGIC [45] iterative multivariate statistical analysis and multi-reference alignment analysis (MSA-MRA). Overlapping particles or dust were removed and only those classes belonging to properly assembled complexes were kept. A new stack was generated with all the particles within these classes and subjected to five final rounds of MSA-MRA.

## Supporting Information

File S1 **File S1 includes Figures S1, S2 and S3. File S1 also includes Tables S1, S2 and S3.**
(DOCX)
