# Supplementary material for: Human-Like Eukaryotic Translation Initiation Factor 3 from Neurospora crassa
Source: PLoS One. 2013 Nov 8;8(11):e78715. doi: 10.1371/journal.pone.0078715 (PMC3826745; doi:10.1371/journal.pone.0078715)
Supplement: File S1 — includes Figures S1, S2 and S3. File S1 also includes Tables S1, S2 and S3. (DOCX) [file pone.0078715.s001.docx]

**Supplemental Data**

**Figure S1**


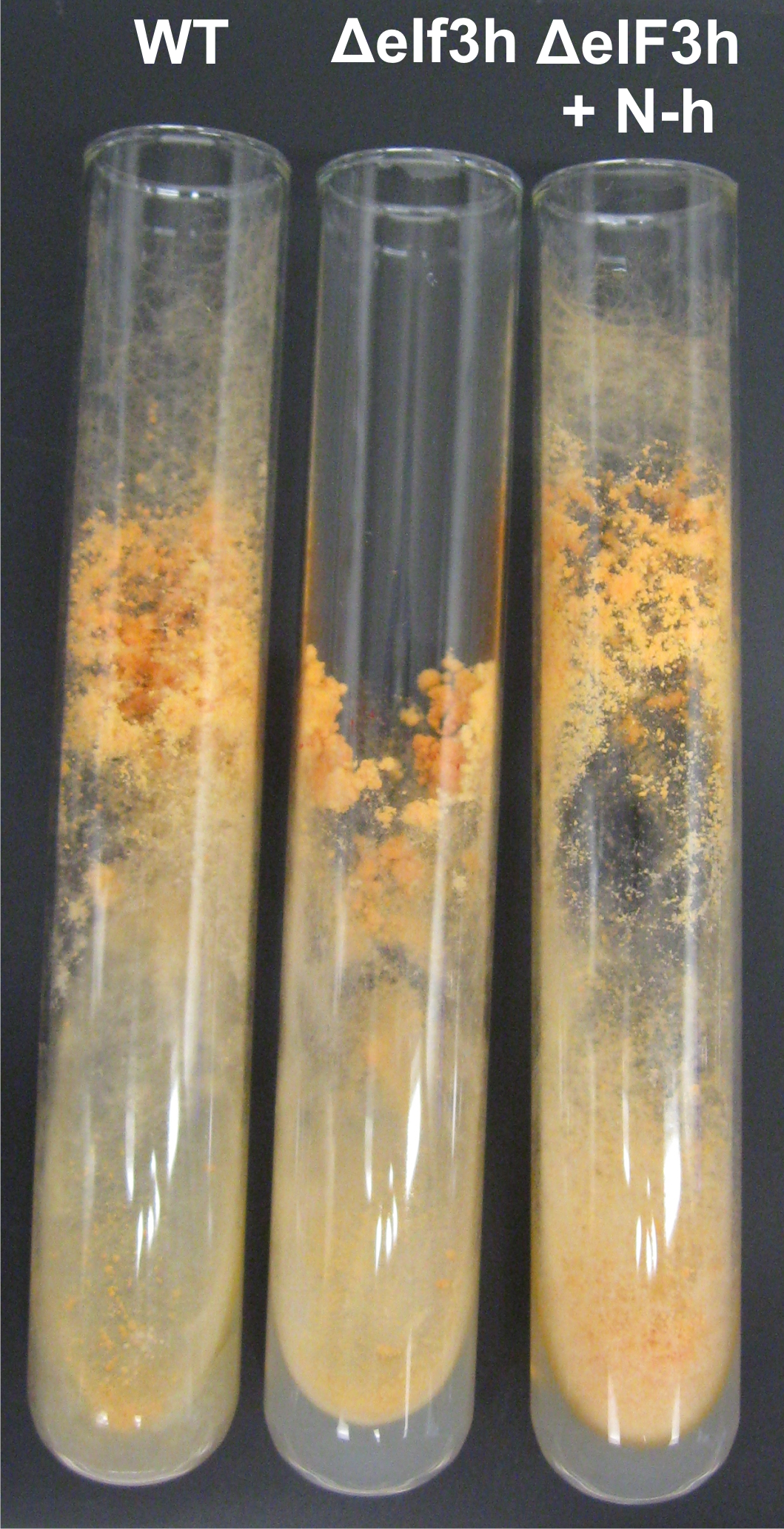


**Figure S1: The eIF3h knock-out in *N. crassa* causes developmental defects in aerial hyphe and conidia.**

Slants of *N. crassa* wild-type (WT), eIF3h knockout (ΔeIF3h) and the eIF3h knockout strains complemented with the N–terminally tagged eIF3h (ΔeIF3h +N-h) grown on Vogel’s MM 2%sucrose, 2% agar.**Figure S2**


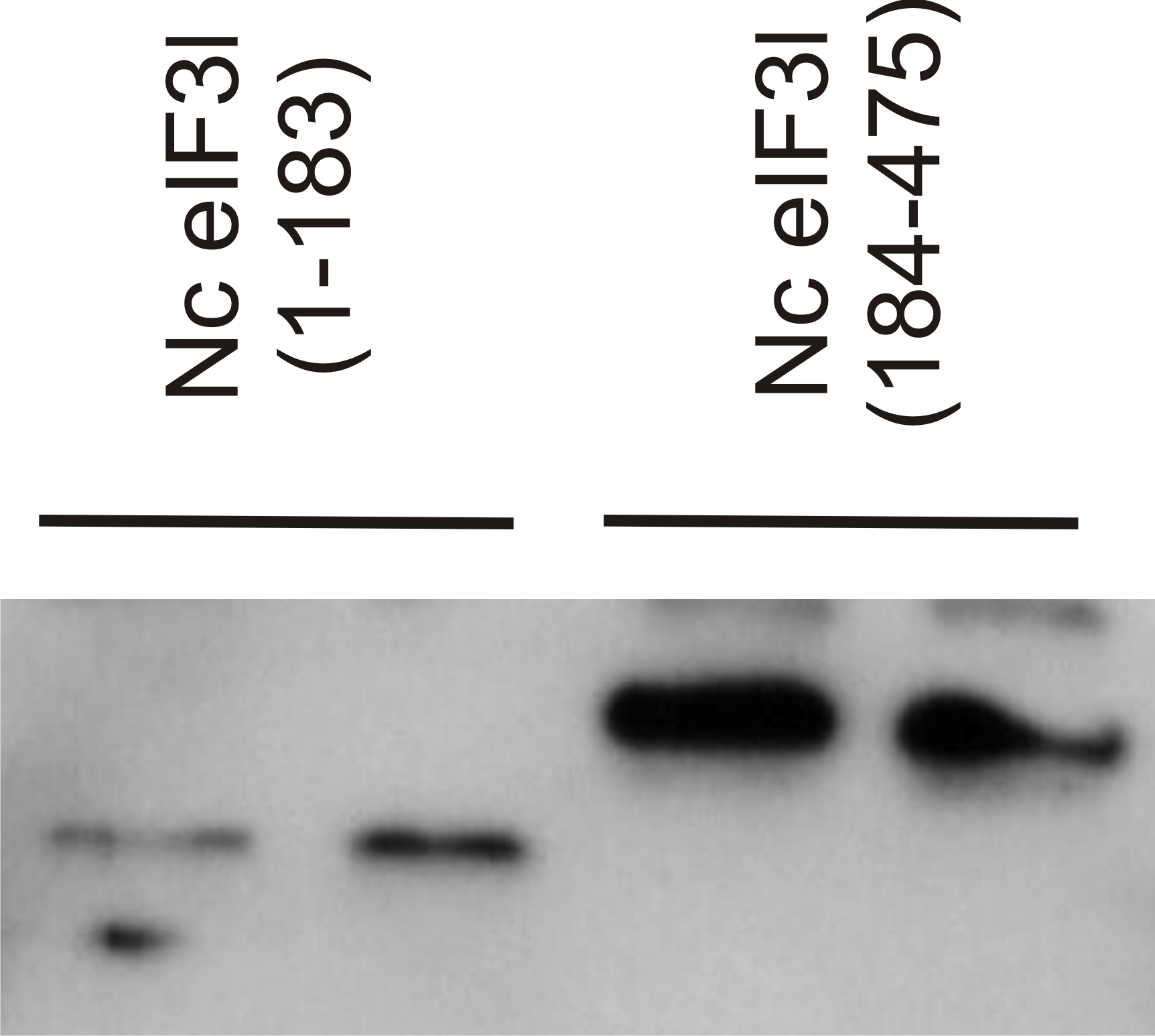


**Figure S2: The N-terminus of eIF3l is responsible for its *in vivo* instability.**

Anti-FLAG Western blot of immunoprecipitated N-terminally FLAG tagged eIF3l truncations from *N. crassa* lysates from equivalent amounts of biomass. Each truncation is labeled over the lanes and consists of two biological replicates. Truncation 1-183 consists of the N-terminal region of eIF3l. Truncation 184-475 consists of the TPR region of eIF3l.

**Figure S3**


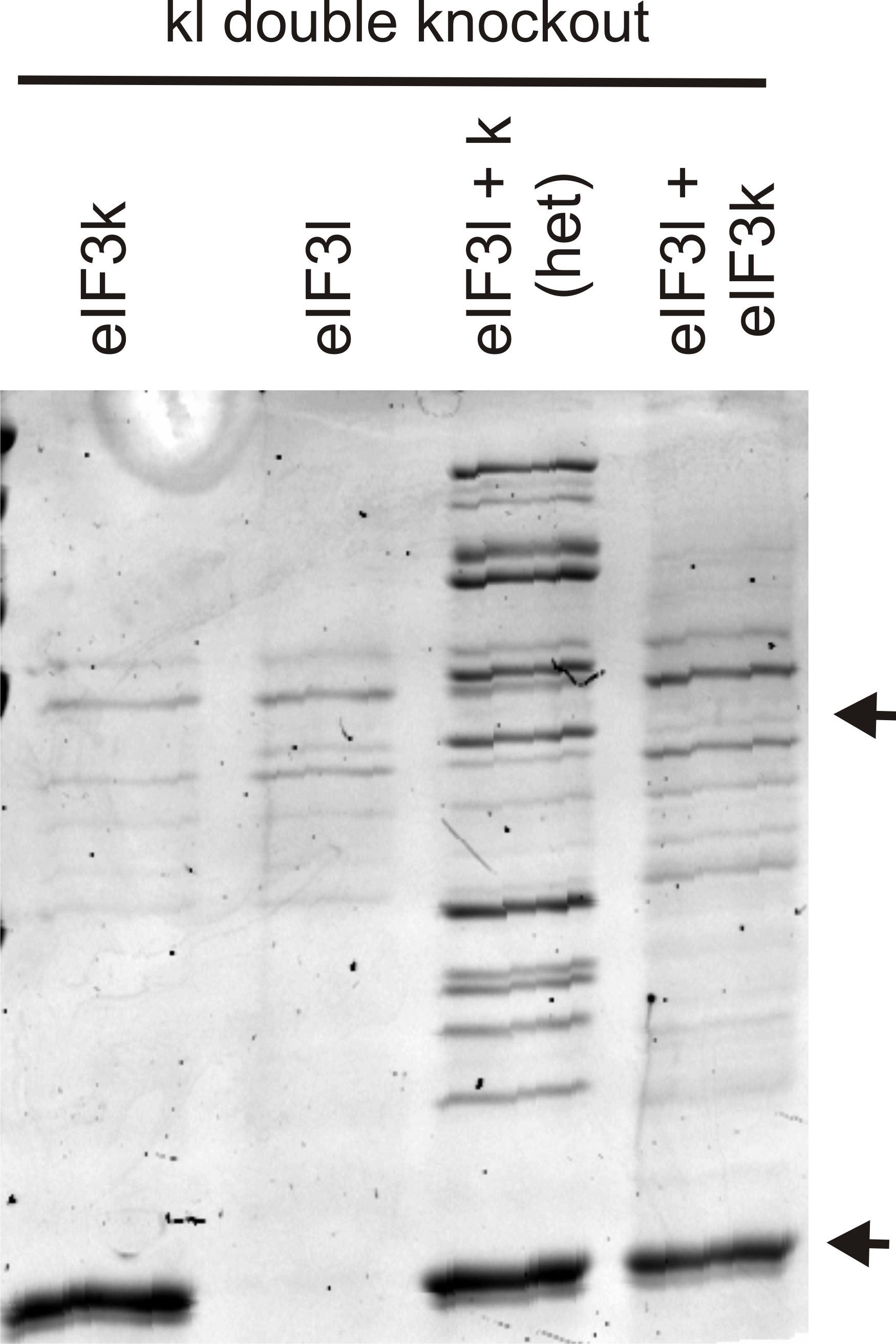


**Figure S3: The *N. crassa* eIF3 dodecamer cannot be reconstituted from lystates of ΔeIF3k and ΔeIF3l strains.**

Affinity purification experiment analogous to Figure 4A. Lane 4 (eIF3l + eIF3k) is an affinity purification using mixed and 5X concentrated eluates from Lanes 1 and 2. Arrows highlight the locations where subunits k and l are expected to migrate, other bands are non-specific contaminants.

**Table S1: List of *Neurospora crassa* strains**

| **Strains obtained from the FGSC** | | | | | | | | |
| --- | --- | --- | --- | --- | --- | --- | --- | --- |
| *Strain* | *Genotype* | *FGSC#* |  | | | *Strain* | *Genotype* | *FGSC#* |
| Wild-type | *mat a* | 2484 |  | | | ΔeIF3h | *mat A* | 20246 |
| fluffy | *A: fl* | 4960 |  | | | ΔeIF3h | *mat a* | 20245 |
| fluffy | *a: fl* | 4961 |  | | | ΔeIF3i | *mat a* | 15693 |
| N623 | *mat A his-3* | 6103 |  | | | ΔeIF3j | *mat A* | 19351 |
| ΔeIF3a | *mat a* | 22127 |  | | | ΔeIF3j | *mat a* | 19350 |
| ΔeIF3c | *mat a* | 20914 |  | | | ΔeIF3k | *mat A* | 18546 |
| ΔeIF3d | *mat a* | 15494 |  | | | ΔeIF3k | *mat a* | 18547 |
| ΔeIF3e | *mat a* | 20906 |  | | | ΔeIF3l | *mat A* | 12947 |
| ΔeIF3f | *mat a* | 16510 |  | | | ΔeIF3l | *mat a* | 12946 |
| ΔeIF3g | *mat a* | 22128 |  | | | ΔeIF3m | *mat a* | 13595 |
|  |  |  |  | | |  |  |  |
| **Strains created in this study** | | | | | | | | |
| *Strain* | *Genotype* | | |  | *Strain* | | *Genotype* | |
| ΔeIF3e his-3 | *mat a his-3* | | |  | ΔeIF3k +V | | *mat a his-3(pccg1::N-FLAG::HAT)* | |
| ΔeIF3hk | *mat A* | | |  | ΔeIF3k +N-k | | *mat a his-3(pccg1::N-FLAG::HAT::Nc-eIF3k)* | |
| ΔeIF3h his-3 | *mat A his-3* | | |  | ΔeIF3k +C-k | | *mat a his-3(pccg1::C-Gly::HAT::FLAG::Nc-eIF3k)* | |
| ΔeIF3j his-3 | *mat a his-3* | | |  | ΔeIF3l +V | | *mat A his-3(pccg1::N-FLAG::HAT)* | |
| ΔeIF3k | *mat A* | | |  | ΔeIF3l +N-l | | *mat A his-3(pccg1::N-FLAG::HAT::Nc-eIF3l)* | |
| ΔeIF3k his-3 | *mat A his-3* | | |  | ΔeIF3l +C-l | | *mat A his-3(pccg1::C-Gly::HAT::FLAG::Nc-eIF3l)* | |
| ΔeIF3l | *mat A his-3* | | |  | ΔeIF3kl his-3 | | *mat A his-3* | |
| ΔeIF3l | *mat a* | | |  | ΔeIF3kl | | *mat A his-3(pccg1::N-FLAG::HAT)* | |
| ΔeIF3h +V | *mat A his-3(pccg1::N-FLAG::HAT)* | | |  | ΔeIF3kl +N-k | | *mat A his-3(pccg1::N-FLAG::HAT::Nc-eIF3k)* | |
| ΔeIF3h +N-h | *mat A his-3(pccg1::N- FLAG::HAT::Nc-eIF3h)* | | |  | ΔeIF3kl +N-l | | *mat A his-3(pccg1::N-FLAG::HAT::Nc-eIF3l)* | |
| ΔeIF3h +C-h | *mat a his-3(pccg1::C-Gly::HAT::FLAG::Nc-eIF3h)* | | |  | ΔeIF3j +N-j | | *mat a his-3(pccg1::N-FLAG::HAT::Nc-eIF3j)* | |
| ΔeIF3j +V | *mat a his-3(pccg1::N-FLAG::HAT)* | | |  | ΔeIF3k +V | | *mat a his-3(pccg1::N-FLAG::HAT)* | |
| ΔeIF3j +C-J | *mat a his-3(pccg1::C-Gly::HAT::FLAG::Nc-eIF3j)* | | |  | ΔeIF3jl | |  | |
| ΔeIF3hl +N-h | *mat A his-3(pccg1::N- FLAG::HAT::Nc-eIF3h)* | | |  | ΔeIF3hk +N-k | | *mat a his-3(pccg1::N-FLAG::HAT::Nc-eIF3k)* | |
| ΔeIF3l +C-l (1-183) | *mat A his-3(pccg1::C-Gly::HAT::FLAG::Nc-eIF3l)* | | |  | ΔeIF3l +C-l (184-475) | | *mat A his-3(pccg1::C-Gly::HAT::FLAG::Nc-eIF3l)* | |

**Table S2: Relative linear growth rates for *N. crassa* eIF3 knock-out strains**

|  | | | Linear growth relative to wild-type | | | | | | |  |
| --- | --- | --- | --- | --- | --- | --- | --- | --- | --- | --- |
|  | | | MM | +/- | | H_2_O agar | | +/- | | H_2_0/MM* |
| Wild-type | | | 1 | 0.01 | | 1 | | 0.01 | | 0.63 |
| ΔeIF3e | | | 0.57 | 0.01 | | 0.67 | | 0.01 | | 0.72 |
| ΔeIF3h | | | 0.77 | 0.01 | | 0.66 | | 0.01 | | 0.52 |
| ΔeIF3j | | | 0.65 | 0.02 | | 0.60 | | 0.02 | | 0.60 |
| ΔeIF3k | | | 0.97 | 0.01 | | 0.85 | | 0.01 | | 0.51 |
| ΔeIF3l | | | 0.95 | 0.01 | | 0.85 | | 0.01 | | 0.54 |
| ΔeIF3kl | | | 0.98 | 0.00 | | 0.82 | | 0.00 | | 0.52 |
| ΔeIF3hk | | | 0.76 | 0.01 | | 0.69 | | 0.01 | | 0.57 |
| ΔeIF3jl | | | 0.60 | 0.02 | | 0.60 | | 0.02 | | 0.74 |
|  |  |  | | |  | |  | |  |  |

*Calculated using raw linear growth values

**Table S3: List of proteins that co-purify with eIF3j from *N. crassa***

| **Uniprot Name** | **Full protein name** | | **MW (kDa)** | **pI** | **Peptides** |
| --- | --- | --- | --- | --- | --- |
| *Translation factors* | | | | | |
| EIF3J_NEUCR | Eukaryotic translation initiation factor 3 subunit J | | 30.2 | 4.9 | 141 |
| EF1A_NEUCR | Elongation factor 1 alpha | | 49.6 | 9.5 | 33 |
| EIF3A_NEUCR | Eukaryotic translation initiation factor 3 subunit A | | 114.4 | 9.8 | 14 |
| EIF3B_NEUCR | Eukaryotic translation initiation factor 3 subunit B | | 85.5 | 4.8 | 11 |
| EIF3C_NEUCR | Eukaryotic translation initiation factor 3 subunit C | | 98.3 | 4.9 | 11 |
| EF2_NEUCR | Elongation factor 2 | | 93.2 | 6.2 | 10 |
| Q7Z8V4_NEUCR | Translation elongation factor 1 alpha Fragment | | 33.3 | 8.2 | 8 |
| EIF3I_NEUCR | Eukaryotic translation initiation factor 3 subunit I | | 38.7 | 6.2 | 6 |
| EIF3D_NEUCR | Eukaryotic translation initiation factor 3 subunit D | | 64.9 | 5.0 | 5 |
| EIF3K_NEUCR | Eukaryotic translation initiation factor 3 subunit K | | 26.7 | 4.3 | 5 |
| EIF3F_NEUCR | Eukaryotic translation initiation factor 3 subunit F | | 39.6 | 4.7 | 4 |
| EIF3H_NEUCR | Eukaryotic translation initiation factor 3 subunit H | | 40.3 | 6.1 | 4 |
| EIF3L_NEUCR | Eukaryotic translation initiation factor 3 subunit L | | 54.4 | 4.9 | 4 |
| *Ribosomal Proteins* | | | | | |
| Q7RV52_NEUCR | | Cytoplasmic ribosomal protein subunit S3 | 28.6 | 9.0 | 12 |
| Q9C2H7_NEUCR | | 60S ribosomal protein L4 A | 38.7 | 11.2 | 11 |
| RS5_NEUCR | | 40S ribosomal protein S5 | 23.6 | 9.5 | 10 |
| Q7RVQ9_NEUCR | | 60S ribosomal protein L2 | 27.3 | 11.4 | 8 |
| Q7S7F0_NEUCR | | 60S ribosomal protein L10 | 25.3 | 10.7 | 7 |
| Q7S630_NEUCR | | 60S ribosomal protein L8 | 29.3 | 10.7 | 6 |
| RSSA_NEUCR | | 40S ribosomal protein S0 | 31.4 | 4.6 | 6 |
| Q7S709_NEUCR | | 60S ribosomal protein L13 | 23.8 | 11.3 | 5 |
| Q7SD62_NEUCR | | 60S ribosomal protein L20 | 20.3 | 10.9 | 5 |
| Q7SE03_NEUCR | | 40S ribosomal protein S4 | 29.5 | 10.5 | 5 |
| RS7_NEUCR | | 40S ribosomal protein S7 | 22.8 | 10.6 | 5 |
| Q7SHJ9_NEUCR | | 60S ribosomal protein L23 | 14.6 | 10.7 | 4 |
| RL10A_NEUCR | | 60S ribosomal protein L10a | 24.1 | 10.3 | 4 |
| RS25_NEUCR | | 40S ribosomal protein S25 | 10.7 | 10.4 | 4 |
| RS3A_NEUCR | | 40S ribosomal protein S1 | 29.0 | 10.4 | 4 |
| *Other Proteins* | | | | | |
| ATPA_NEUCR | | ATP synthase subunit alpha mitochondrial | 59.4 | 9.2 | 25 |
| ACT_NEUCR | | Actin | 41.5 | 5.3 | 19 |
| Q7RW06_NEUCR | | Heat shock protein SSB1 | 66.0 | 5.0 | 16 |
| PGK_NEUCR | | Phosphoglycerate kinase | 45.0 | 6.1 | 13 |
| Q7SD02_NEUCR | | Putative uncharacterized protein | 68.2 | 8.3 | 12 |
| ADT_NEUCR | | ADP ATP carrier protein | 33.8 | 10.1 | 11 |
| CSK2A_NEUCR | | Casein kinase II subunit alpha | 39.6 | 6.8 | 10 |
| Q1K7A2_NEUCR | | Putative uncharacterized protein | 66.8 | 4.9 | 9 |
| TBB_NEUCR | | Tubulin beta chain | 49.8 | 4.6 | 9 |
| Q12642_NEUCR | | S adenosylmethionine synthase Fragment | 39.1 | 5.3 | 8 |
| CYPH_NEUCR | | Isoform Cytosolic of Peptidyl prolyl cis trans isomerase mitochondrial | 19.5 | 8.7 | 7 |
| Q7RVX2_NEUCR | | Heat shock 70 kDa protein mitochondrial | 72.6 | 5.6 | 6 |
| Q7SEL5_NEUCR | | UTP glucose 1 phosphate uridylyltransferase | 58.1 | 6.6 | 6 |
| MPPB_NEUCR | | Mitochondrial processing peptidase subunit beta | 52.5 | 5.5 | 5 |
| ALF_NEUCR | | Fructose bisphosphate aldolase | 39.8 | 5.2 | 4 |
| GRP78_NEUCR | | 78 kDa glucose regulated protein homolog | 72.2 | 4.8 | 4 |
| Q7SBB3_NEUCR | | Predicted protein | 15.9 | 11.6 | 4 |
